# Use of complementary and alternative medicine for reducing fear of cancer recurrence among cancer survivors: Does it work?

**DOI:** 10.1016/j.apjon.2023.100278

**Published:** 2023-07-25

**Authors:** Heliang Wu, Adnan Rashid Aziz, Mahlagha Dehghan, Leyla Ahmadi Lari, Rasmieh Al-Amer, Mohammad Ali Zakeri

**Affiliations:** a Hainan Vocational University of Science and Technology, Haikou, Hainan, China; b Nursing Research Center, Kerman University of Medical Sciences, Kerman, Iran; c College of Nursing, Al Kitab University, Kirkuk, Iraq; d M.Sc of Critical Care Nursing, Department of Nursing, School of Nursing, Larestan University of Medical Sciences, Larestan, Iran; e Isra University of Jordan, School of Nursing, Amman, Jordan; f Western Sydney University, School of Nursing and Midwifery, New South Wales (NSW), Australia; g Non-Communicable Diseases Research Center, Rafsanjan University of Medical Sciences, Rafsanjan, Iran

**Keywords:** Alternative medicine, Cancer survivor, Complementary, Fear of cancer recurrence

## Abstract

**Objective:**

Fear of cancer recurrence among cancer survivors is a psychosocial concern that affects recovery and quality of life. They use complementary and alternative medicine to prevent the side effects of drugs and relieve anxiety and fear of cancer recurrence. This study aimed to examine the correlation between the use of complementary and alternative medicine and the fear of cancer recurrence in cancer survivors.

**Methods:**

This cross-sectional descriptive correlational study enrolled 280 cancer survivors referred to oncology centers and medical offices in Kerman using convenience sampling. The research tools included complementary and alternative medicine questionnaire and the fear of cancer recurrence inventory. IBM SPSS Statistics version 25 was used to analyze the data.

**Results:**

The study findings revealed that 78.2% of the participants used at least one type of complementary and alternative medicine in the last year; 71.8% used medicinal herbs, 19.6% used nutritional supplements, 7.5% used relaxation and meditation, 7.1% used dry cupping, and 5.7% used wet cupping. The mean score of fear of cancer recurrence was 80.72 ​± ​18.46, which was almost near the midpoint of the inventory score (84). The fear of cancer recurrence and its dimensions did not differ between users and nonusers of complementary and alternative medicine.

**Conclusions:**

Our results suggested that most of the survivors used at least one type of complementary and alternative medicine in the past year, and medicinal herbs and nutritional supplements were the most used types. Patients with cancer must be aware of the effects of different kinds of complementary and alternative medicine. A moderate level in the mean score of fear of cancer recurrence was found, and no difference was noted between users and nonusers of complementary and alternative medicine. Health managers and planners should conduct effective psychological interventions and strategies to minimize the fear of cancer recurrence among cancer survivors.

## Introduction

Cancer has many physical and psychological consequences. Estimates have indicated that more than 1.7 million people will live with cancer, of whom, more than 600,000 will die in 2028.^1^ Many patients with cancer survive due to timely diagnosis, access to healthcare services, and improved support systems.^2^ Currently, 15.5 million cancer survivors live in the USA, and this number is projected to rise to 20.3 million in 2026.^3^ The Canadian Cancer Statistics reported that more than 220,400 new cancer cases were documented in 2019, 63% of which have at least 5 years of survival after diagnosis.^4^ Owing to advances in cancer diagnosis and treatment, two-thirds of those diagnosed with aggressive cancer can live for > 5 years. With the increase in the cancer survival rate, mental health concerns represent a large part of the unmet needs of the survivors, and anxiety and depression, as well as various psychological aspects related to cancer, are prevalent among cancer survivors.^5^ They usually suffer from persistent physical symptoms related to treatment, such as but not limited to pain, fatigue, and temporary amnesia. In addition, they experience a wide range of psychological difficulties and distress due to fear of recurrence and changes in family relationships.^4^ For example, they experience high levels of psychological stress because cancer metastasizes, and recurrence could take place in some cases.^6^ Patients with chronic illness suffer from disease-related fear, which is referred to as the fear of disease progression or recurrence.^7^ Patients with high levels of fear of cancer recurrence experience higher anxiety and excessive distress, which in turn impedes successful psychological adjustment in patients with cancer.^8^

Fear of cancer recurrence is one of the five major concerns of cancer survivors on a global scale.^9^ Shay et al. reported that 85.2% of cancer survivors had high levels of fear of cancer recurrence.^10^ Patients in the active stage of the disease feel that the disease is a threat and is uncontrollable and reported higher levels of fear of recurrence.^11^^,^^12^ Cancer survivors use nonpharmacological methods to relieve anxiety and stress because they are not comfortable with the side effects of the psychiaty-related disease. Thus, complementary and alternative medicine (CAM) is a common method in addition to modern pharmaceutical treatments to control symptoms and improve the condition of patients with chronic illness.^13^^,^^14^

According to the World Health Organization, CAM is viewed as knowledge, skills, and practices based on indigenous theories, beliefs, and experiences in different cultures to maintain health and prevent, improve, or treat physical and mental problems. CAM includes more than 1800 types, such as herbal medicines, vitamins, probiotics, psychotherapy, acupuncture, acupressure, and cupping.^15^ Cancer survivors use CAM to deal with pain, insomnia, persistent psychological distress and side effects of drugs, increase treatment options, manage symptoms, and improve and strengthen the immune system.^16^

In order to find relevant articles across various databases, we used keywords such as alternative medicine, complementary medicine, fear of cancer recurrence, cancer survivors, and cancer. We have curated a selection of relevant articles sourced from the retrieved databases, a few of which are outlined as follows. Holmes et al. found that cancer survivors used 1–18 types of CAM; the most commonly used types were massage (82%), acupuncture (64%), and vitamins and minerals (64%).^17^ Qureshi et al. indicated that 75% of cancer survivors used CAM; of whom, 21% used psychotherapy, and 11% used energy therapy. Cancer survivors mostly used massage, vitamin B12, breathing and relaxation exercises, mindfulness-based stress reduction, antioxidants, multivitamins, meditation, yoga, visualization and guided imagery, and calcium.^18^ Gansler et al (2019) found that 5 (0.4%) participants used CAM without evidence only for recurrence prevention, and 181 (14.8%) participants had recurrence prevention as their only or one of the reasons for using CAM without evidence for recurrence prevention.^19^ Sohl et al. demonstrated that 18.9% of their patients used complementary health approaches, a branch of CAM, to prevent cancer recurrence.^20^

The number of cancer survivors is increasing. They suffer from various persistent physical and psychological symptoms, such as anxiety and fear of recurrence; thus, they are using CAM to improve and reduce physical and psychological symptoms. Informing the literature about the fear of recurrence would enable healthcare providers to plan and tailor interventions and provide individualized services for cancer survivors.^18^^,^^20^ As studies in this field are limited, this study aimed to inform the literature and evaluate the types of CAM used by patients with cancer and examine the levels of fear in these patients. In addition, this study examined the association between the use of CAM and the fear of recurrence among cancer survivors in southern Iran.

This study is guided by the following research questions.

1. What are the reasons and types of CAM used by patients with cancer?
2. What are the levels of fear of cancer recurrence among cancer survivors?
3. Is there an association between the use of CAM and different aspects of fear of cancer recurrence among cancer survivors?
4. Is there an association between the use of CAM and background information among cancer survivors?
5. Do users of CAM have higher levels of fear than nonusers?

## Methods

### Study design and setting

This descriptive and analytical cross-sectional study was conducted to determine the association between the use of CAM and the fear of cancer recurrence among cancer survivors in Iran. The study enrolled all cancer survivors referred to oncology centers and medical offices in Kerman. Data were collected from January 1 to August 31, 2021.

### Sampling and sample size

After the ethical approval was received, a convenience-sampling approach was used to select participants from Bahonar Hospital, Javad Al-A'meh Center, and medical offices (three oncologists and hematologists in Kerman). The inclusion criteria were as follows: willingness to participate in the study; diagnosis, treatment, and full recovery from cancer; being educated; and absence of hearing, visual, and cognitive disabilities as indicated by the patients and their medical records. The exclusion criteria were as follows: participants who were unable to complete the questionnaires because of their cognitive problems, unfamiliarity with the Persian language, and incomplete questionnaires.

As we found no similar study on the correlation between CAM and fear of cancer recurrence, we used Cochran's formula for an infinite population to estimate the sample size (*Z* ​= ​1.96, *d* ​= ​0.065, *n* ​= ​227). According to the dropout probability, 300 questionnaires were distributed; however, only 280 participants completed the questionnaire; thus, the response rate was 93.33%.

### Data collection

Initially, the heads of the healthcare team members at the participating hospitals and centers were approached, they received a detailed explanation of the purpose of this study, and they were asked to facilitate the data-collection process for this research. After agreeing to help in the data collection, potential participants were approached, and they received an explanation of the study's purpose. Those who agreed to participate in the study were screened for their eligibility. Those who fulfilled the inclusion criteria for this study were provided with a detailed explanation of the study and were informed about their rights of leaving the study at any time without any penalties or justifications. Then, they were asked to provide informed consent. The participants were handed the questionnaires that included the demographic information questionnaire, CAM questionnaire, and fear of cancer recurrence inventory (FCRI), and they were asked to return the survey questionnaire to the first researcher who was available during data collection. Those who asked for help, such as uneducated patients, received help from the first researcher to complete the questionnaires.

### Study measures

#### Sociodemographic and clinical questionnaire

The sociodemographic (7 items) and clinical information (9 items) questionnaire was developed by the authors and was based on existing literature. Sociodemographic information included age, sex, marital status, education level, occupation, income, and place of residence. Clinical information of patients included the type of cancer, duration of cancer recovery, reception of maintenance therapy, and a history of addiction, diabetes, hypertension, cardiovascular disease, other chronic illnesses, and infection with coronavirus.

#### Complementary and alternative medicine questionnaire

Dehghan et al. developed this 10-item questionnaire to measure the use of different types of CAM (such as herbal medicines, wet cupping, dry cupping, massage, acupuncture, acupressure, homeopathy, and relaxation techniques, such as yoga and prayer). The items were scored based on a 7-point Likert scale (from never/rarely [0] to every day [6]). Reasons to use CAM were to reduce physical symptoms, reduce anxiety and stress, and others. The participants were asked a yes/no question if they should seek help from a doctor before taking CAM. Ten faculty members of the Razi School of Nursing and Midwifery in Kerman determined the validity of the questionnaire. The experts checked the questionnaire for the relevance, comprehensiveness, and comprehensibility of the items, response options, and instructions. The experts checked and scored the relevancy of each item using a four-point Likert scale to calculate the content-validity index (0.96). For internal consistency, 20 participants completed the questionnaire, and Cronbach's alpha coefficient was 0.85.^15^

#### Fear of cancer recurrence inventory

Simrad et al. developed this 42-item scale based on a Likert scale ranging from 0 to 4 (never, 0; rarely, 1; sometimes, 2; very often, 3; always, 4). In this questionnaire, “I believe that I have been treated” and “My illness will not return” have reverse scores (never, 4; rarely, 3; sometimes, 2; very often, 1; always, 0). The lowest and highest scores on this scale are 0 and 168, respectively. This questionnaire assesses seven dimensions, namely, triggers (8 items), severity (9 items), psychological distress (4 items), coping strategies (9 items), functioning impairments (6 items), insight (3 items), and reassurance (3 items), with higher scores reflecting higher levels of fear of cancer recurrence. The internal consistency and retest validity of the original French version were 0.75 and 0.58, respectively.^21^ According to Bateti et al., in the Iranian population, Cronbach's alpha coefficient was 0.86, the test–retest reliability was 0.96, and the validity of triggers, severity, psychological distress, functioning impairments, insight, reassurance, coping strategies, and overall questionnaire were 0.82, 0.84, 0.80, 0.85, 0.77, 0.79, 0.78, and 0.80, respectively.^22^ In the present study, the Cronbach's alpha of FCRI was 0.88.

### Data analysis

Data were analyzed using IBM SPSS Statistics for Windows version 25 (IBM Corp., Armonk, NY, USA) and descriptive and inferential statistical methods. Descriptive statistics, including frequency, percentage, mean, and standard deviation, were used to describe demographic characteristics and mean scores. The independent *t*-test was used to determine the scores of FCRI and its dimensions according to users and nonusers of CAM and CDAS. The Chi-square test was used to assess the association between study variables and the use of CAM (yes/no). A significance level of 0.05 was considered.

### Ethical considerations

Sampling was started after receiving ethical approval (No. IR.RUMS.REC.1399.237) from the Ethics Committee of Rafsanjan University of Medical Sciences and obtaining informed consent from the participants. The participants were informed about the objectives of the study, confidentiality, and anonymity of information, and that they were free to complete the questionnaire.

## Results

### Participants’ demographic and clinical characteristics

The study enrolled 280 participants, with a mean age of 53.24 ​± ​11.37 (min, 21; max, 84) years. Moreover, 81.4% of the participants were women (*n* ​= ​228), 90.4% were married (*n* ​= ​253), 15.0% were uneducated (*n* ​= ​42), and 60.0% were unemployed (*n* ​= ​168). In addition, 66.4% (*n* ​= ​186) of the participants had a monthly income of more than two million Toman; (1 Toman ​= ​0.00002791 USD) (Table 1). Furthermore, 64.6% of the participants (*n* ​= ​181) had breast cancer, and 60.0% (*n* ​= ​168) experienced a > 1-year remission from cancer. Approximately 19.0% of the participants were infected with coronavirus (Table 2).

**Table 1:** Participants’ demographic characteristics and their association with complementary and alternative medicine use among the participants (*N* = 280).

Variable	*n* (%)	CAM user		χ^2^ (*P* value)
		Yes (*n* = 219), *n* (%)	No (*n* = 61), *n* (%)	
Age, years				
≤ 40	36 (12.9)	26 (11.9)	10 (16.4)	0.93 (0.82)
41–50	79 (28.2)	63 (28.8)	16 (26.2)	
51–60	92 (32.9)	72 (32.9)	20 (32.8)	
> 60	73 (26.1)	58 (26.5)	15 (24.6)	
Gender				
Female	228 (81.4)	173 (79.0)	55 (90.2)	3.94 (0.047)
Male	52 (18.6)	46 (21.0)	6 (9.8)	
Marital status				
Married	253 (90.4)	200 (91.3)	53 (86.9)	1.14 (0.57)
Single	16 (5.7)	11 (5.0)	5 (8.2)	
Divorced/widow (er)	11 (3.9)	8 (3.7)	3 (4.9)	
Education level				
Uneducated	42 (15.0)	26 (11.9)	16 (26.2)	12.17 (0.007)
Under diploma	87 (31.1)	77 (35.2)	10 (16.4)	
Diploma	93 (33.2)	71 (32.4)	22 (36.1)	
Academic	58 (20.7)	45 (20.5)	13 (21.3)	
Job				
Employed	68 (24.3)	56 (25.6)	12 (19.7)	1.13 (0.58)
Retired	44 (15.7)	35 (16.0)	9 (14.8)	
Unemployed	168 (60.0)	128 (58.4)	40 (65.6)	
Monthly income (million Toman)				
Less than one	38 (13.6)	25 (11.4)	13 (21.3)	4.47 (0.11)
1–2	56 (20.0)	43 (19.6)	13 (21.3)	
> 2	186 (66.4)	151 (68.9)	35 (57.4)	
Living place				
Kerman city	135 (48.2)	108 (49.3)	27 (44.3)	2.35 (0.50)
Villages around Kerman	35 (12.5)	28 (12.8)	7 (11.5)	
Cities around Kerman	100 (35.7)	77 (35.2)	23 (37.7)	
Other cities	10 (3.6)	6 (2.7)	4 (6.6)	
Insurance				
Yes	271 (96.8)	212 (96.8)	59 (96.7)	0.001 (0.97)
No	9 (3.2)	7 (3.2)	2 (3.3)	

CAM, complementary and alternative medicine.

**Table 2:** Participants’ clinical characteristics and their association with complementary and alternative medicines use among the participants (*N* = 280).

Variable	*n* (%)	CAM user		χ^2^ (*P* value)
		Yes (*n* = 219), *n* (%)	No (*n* = 61), *n* (%)	
Type of cancer				
Breast	181 (64.6)	139 (63.5)	42 (68.9)	1.83 (0.61)
Gastrointestinal	36 (12.9)	31 (14.1)	5 (8.2)	
Genital	31 (11.1)	25 (11.4)	6 (9.8)	
Others	32 (11.4)	24 (11.0)	8 (13.1)	
Duration of cancer remission, years				
≤ 1.00	112 (40.0)	85 (38.8)	27 (44.3)	1.26 (0.74)
1.01–3.00	84 (30.0)	66 (30.1)	18 (29.5)	
3.01–5.00	40 (14.3)	31 (14.2)	9 (14.8)	
> 5.00	44 (15.7)	37 (16.9)	7 (11.5)	
Receiving maintenance treatment				
Yes	126 (45.0)	96 (43.8)	30 (49.2)	0.55 (0.46)
No	154 (55.0)	123 (56.2)	31 (50.8)	
History of addiction				
Yes	32 (11.4)	30 (13.7)	2 (3.3)	5.12 (0.02)
No	248 (88.6)	189 (86.3)	59 (96.7)	
History of diabetes				
Yes	45 (16.1)	34 (15.5)	11 (18.0)	0.22 (0.64)
No	235 (83.9)	185 (84.5)	50 (82.0)	
History of hypertension				
Yes	89 (31.8)	75 (34.2)	14 (23.0)	2.81 (0.09)
No	191 (68.2)	144 (65.8)	47 (77.0)	
History of cardiovascular disease				
Yes	38 (13.6)	34 (15.5)	4 (6.6)	3.27 (0.07)
No	242 (86.4)	185 (84.5)	57 (93.4)	
History of other chronic disease				
Yes	28 (10.0)	22 (10.0)	6 (9.8)	0.002 (0.96)
No	252 (90.0)	197 (90.0)	55 (90.2)	
Infected with coronavirus				
Yes	53 (18.9)	45 (20.5)	8 (13.1)	1.72 (0.19)
No	227 (81.1)	174 (79.5)	53 (86.9)	

CAM, complementary and alternative medicine.

### Use of CAM and FCRI among cancer survivors

All participants used prayer as a type of complementary approach to escape fear; hence, we believe that this item has reached a ceiling effect, so this was excluded from further analysis. As illustrated in Table 3, 78.2% of the participants used at least one type of CAM in the last year (*n* ​= ​219; 95% confidence interval [CI], 73.6-82.9). Regardless of prayer, 50.0% of the participants used at least one type of CAM in the last year (*n* ​= ​201, 95% CI, 43.9-55.7). In addition, 21.4% of the individuals used two types of CAM (*n* ​= ​60, 95% CI, 16.8-26.1), 5.0% used three types of CAM (n ​= ​14, 95% CI, 2.5-7.5), 1.4% used four types of CAM (*n* ​= ​4, 95% CI, 0.4-2.9), and 0.4% used five types of CAM in the last year (*n* ​= ​1, 95% CI, 0.0-1.1). Moreover, 71.8% used medicinal herbs, 19.6% used nutritional supplements, 7.5% used relaxation and meditation, 7.1% used dry cupping, 5.7% used wet cupping, 3.2% used massage, and 0.4% used homeopathy (Table 3). None of the participants used acupuncture or acupressure. As regards the use of medicinal herbs, nutritional supplements, dry cupping, and wet cupping, 24.6%, 24.3%, 3.9%, and 3.6% of the participants sought medical advice, respectively.

**Table 3:** The use of complementary and alternative medicines and the reasons for using each type of complementary and alternative medicine among the participants.

Variable	*n* (%)	95% confidence interval of percentage (%)	Reasons for using the CAM methods (*n* [%]^a^)		
			Reducing physical complications of the cancer and its treatment	Reducing stress and anxiety resulting from cancer and its treatment	Others
Medicinal herbs	201 (71.8)	66.8-77.1	61 (41.5)	55 (37.4)	31 (21.1)
Dry cupping	20 (7.1)	4.3-10.4	13 (81.3)	1 (6.3)	2 (12.5)
Wet cupping	16 (5.7)	3.2-8.6	3 (27.3)	2 (18.2)	6 (54.5)
Massage	9 (3.2)	1.1-5.4	4 (50.0)	4 (50.0)	–
Nutritional supplements	55 (19.6)	15.4-24.6	27 (57.4)	–	20 (42.6)
Homeopathy	1 (0.4)	0-1.4	–	1 (100.0)	–
Relaxation and meditation	21 (7.5)	4.6-10.7	2 (11.8)	15 (88.2)	–
Prayer	273 (97.5)	95.7-99.3	14 (7.5)	161 (86.6)	11 (5.9)

CAM, complementary and alternative medicine.

a Missing values, valid percent; Others: strengthening the immune system, decreasing fatigue.

The mean FCRI score was 80.72 ​± ​18.46 (min, 5; max, 168). Table 4 summarizes the mean and standard deviation of the FCRI subscales. The mean score of fear of cancer recurrence was almost near the midpoint of the inventory score (84).

**Table 4:** The fear of cancer recurrence inventory score according to complementary and alternative medicines user and nonuser.

Variable		Mean	SD	CAM user		Independent *t* test (*P* value)
				Yes (Mean/SD)	No (Mean/SD)	
Fear of cancer recurrence inventory	Triggers	14.95	5.85	15.18 (6.00)	14.11 (5.23)	−1.26 (0.21)
	Severity	16.17	7.55	16.29 (7.42)	15.74 (8.05)	−0.50 (0.62)
	Psychological distress	5.77	3.73	5.57 (3.70)	5.41 (3.84)	−0.86 (0.39)
	Coping strategies	23.20	6.49	23.37 (6.50)	22.61 (6.45)	−0.81 (0.42)
	Functioning impairments	10.36	4.65	10.43 (4.83)	10.08 (3.94)	−0.52 (0.60)
	Insight	4.12	3.27	4.18 (3.33)	3.90 (3.05)	−0.59 (0.55)
	Reassurance	6.15	2.02	6.26 (2.02)	5.74 (1.98)	−1.80 (0.07)
	Total	80.72	18.46	81.59 (18.34)	77.59 (18.69)	−1.50 (0.14)

CAM, complementary and alternative medicine; SD, standard deviation.

### Association between the use of CAM and different aspects of FCRI among cancer survivors

FCRI and all its dimensions did not differ between users and nonusers of CAM (Table 4). The FCRI and all its dimensions were also compared between users and nonusers of medicinal herbs. The scores of fear of cancer recurrence, triggers subscale, and reassurance subscale were significantly higher among medicinal herb users than among nonusers (Table 5).

**Table 5:** The fear of cancer recurrence inventory score according to medicinal herbs users and nonusers.

Variable		Medicinal herbs user		Independent *t* test (*P* value)
		Yes (Mean/SD)	No (Mean/SD)	
Fear of cancer recurrence inventory	Triggers	15.70 (5.69)	13.05 (5.86)	−3.47 (0.001)
	Severity	16.28 (7.28)	15.87 (8.24)	−0.41 (0.68)
	Psychological distress	5.99 (3.66)	5.22 (3.85)	−1.57 (0.12)
	Coping strategies	23.59 (6.59)	22.22 (6.16)	−1.60 (0.11)
	Functioning impairments	10.32 (4.81)	10.44 (4.24)	0.19 (0.85)
	Insight	4.15 (3.31)	4.04 (3.18)	−0.27 (0.79)
	Reassurance	6.32 (2.02)	5.71 (1.97)	−2.29 (0.02)
	Total	82.36 (17.90)	76.54 (19.29)	−2.39 (0.02)

SD, standard deviation.

### Association between the use of CAM and background information among cancer survivors

Men more significantly used CAM than women (*P* ​= ​0.047). Uneducated participants significantly used CAM less than educated participants (*P* ​= ​0.007). Participants with addiction significantly used CAM more than those without addiction (*P* ​= ​0.02) (Table 1, Table 2).

## Discussion

This study assessed the association between the use of CAM and the fear of recurrence among cancer survivors in southern Iran. The sample consisted of 280 Iranians attending Bahonar Hospital, Javad Al-A'meh Center, and medical offices. A high level of fear of cancer recurrence among cancer survivors was reported to be associated with psychological distress and daily dysfunction,^23^ and fear of cancer recurrence can play an important role in the use of complementary medicine among cancer survivors.^24^ The study findings suggested that the FCRI and its dimensions did not differ between users and nonusers of CAM, but the FCRI was significantly higher among users of medicinal herbs than among nonusers.

In this study, all participants used prayer as a complementary approach to alleviate fear, which is not surprising, given the religious and indigenous culture of Iran. As a predominantly Muslim country, many Iranians pray three times a day as part of their religious beliefs. Prayer can have a calming effect on a patient's body and soul during illness, helping them become more accepting of their disease and treatment, and better prepared to continue their treatment. These findings are consistent with those of Dehghan et al., who also reported prayer as the most commonly used method among chronic outpatients in their study.^25^

In the present study, the majority of the participants used at least one type of CAM in the previous year, and medicinal herbs, nutritional supplements, meditation, dry cupping, wet cupping, and massage were types of CAM mostly used. In line with our results, John et al demonstrated that approximately 80% of cancer survivors in the USA used vitamins and minerals in the past year.^26^ Rush et al reported that in the USA, approximately one-third of breast cancer survivors used meditation, massage, herbal medicines, and nutritional supplements.^27^ Sanford et al indicated that in the USA, about a quarter of cancer survivors used herbal medicine, massage, and yoga in the previous year.^28^ According to Kim et al., 42% of lymphoma cancer survivors in South Korea used nutritional supplements.^29^ The use of different types of CAM in different studies can be due to the diversity of treatments in geographic areas^30^ and the differences in religious and cultural beliefs.^31^ Alsharif et al found that patients with cancer used CAM because they find it useful for their treatment and recovery.^32^ Patients with cancer use CAM to meet their needs owing to the difficulty of their treatment course and the benefits of complementary medicine. In addition to physical symptoms, complementary medicine affects the mental state of patients; therefore, they must increase their information and skills in using various types of complementary medicine.

The results of the present study revealed that cancer survivors sought medical advice regarding the use of medicinal herbs, nutritional supplements, dry cupping, and wet cupping. Similarly, Sarradon-Eck et al indicated that cancer survivors in France consulted with their general practitioners about the use of CAM.^33^ Alsharif et al found that patients with cancer sought medical advice to gain information about CAM.^32^ Media advertisements of the use of herbal medicines and various nutritional supplements and the confusion of patients were reasons for seeking help from physicians. Sanford et al showed that approximately 30% of the cancer survivors in the USA did not require medical advice regarding the use of CAM because their physicians might discourage them to use complementary medicine^28^; hence, cancer survivors appear to use social networks and communicate with their virtual friends as their main sources of information about CAM, and they rarely sought medical advice regarding use of CAM.

The study results indicated that cancer survivors had moderate levels of fear of cancer recurrence, which was consistent with the results of Dehghan et al in Iran,^34^ Simard et al,^35^ and Meissner et al in Germany.^36^ Fear of cancer recurrence exists in some cancer survivors even years after diagnosis and treatment; thus, early detection of fear of cancer recurrence as well as early psychosocial care may prevent prolonged psychological complications. Cancer survivors should be encouraged to share their thoughts and feelings with healthcare professionals. However, the results of a few studies were inconsistent with our results and reported that cancer survivors reported high levels of fear or cancer recurrence.^9^^,^^10^^,^^37^ These discrepancies with the results of our study could be related to methodological differences, religious practices, sociocultural background of the patients, and use of different tools to assess fear of cancer recurrence. Of note, no consensus is reached on the distinction between mild, moderate, and severe levels of fear of cancer recurrence in different instruments.^38^ The prevalence and intensity of fear of cancer recurrence should be interpreted with caution because the fear of cancer recurrence is not an ongoing experience and occurs in specific situations, such as clinical evaluations or environmental stimuli (such as TV or Internet).^36^ Despite these limitations, our results suggest that physicians and nurses should be concerned and aware of the fear of cancer recurrence. Patients and caregivers’ screening, psychosocial evaluation, and counseling should be of high priority because the fear of cancer recurrence is one of the unmet needs in cancer survivors that limit their quality of life and psychological welfare.

The study results demonstrated that the FCRI and its dimensions did not differ between users and nonusers of CAM, and the FCRI was significantly higher in users of medicinal herbs than in nonusers. Consistent with our results, Carpenter et al reported no difference in FCRI between users and nonusers of CAM in California. However, cancer survivors who used CAM had poor emotional functioning and more medical problems than nonusers.^39^ In Canada, Champagne et al showed no significant association between the level of FCRI and the use of CAM in patients with cancer.^40^ Nonetheless, in Korea, Kim et al found a direct association between the use of CAM and FCRI, with users of complementary medicine experiencing a higher level of fear of recurrence.^24^ Studies reported a wide range of results that showed high levels of controversiality. This can be due to the different methods used to assess fear of cancer recurrence among cancer survivors, and the use of CAM creates psychological stimuli that make survivors think more about cancer recurrence. Cancer survivors, who used complementary medicine, experienced intrusive thoughts related to cancer recurrence compared to nonusers.^21^ This issue is controversial because studies have shown different stances and reports; thus, the present study is highly important because it adds to the existing literature in terms of the psychological status of cancer survivors. However, more studies are needed to present more solid arguments and report findings that support which claim holds stronger evidence.

Healthcare providers including physicians and nurses should be aware of the levels of fear of cancer recurrence in cancer survivors, use different types of CAM, and provide psychological interventions based on the behaviors of survivors. The fear of cancer recurrence may play an important role in the time and duration of using CAM; thus, more longitudinal studies on the association between the use of CAM and the fear of cancer recurrence are needed.

The study results showed that people with higher education levels used more types of CAM than other people. In the USA, Johnson et al reported that cancer survivors with higher education used more types of CAM,^41^ and in Norway, Kristoffersen et al reported a significant association between the use of CAM and higher education.^42^ However, Erku et al demonstrated that patients without academic degrees used complementary medicine more than those with academic degrees^43^; different results could be due to differences in demographic, clinical, and behavioral characteristics.^15^ People with lower levels of education use traditional methods and people with higher levels of education use modern methods, but this public belief needs more investigation.

Our results indicated that people with addiction used more types of complementary medicine, but in the USA, Ojukwu et al found that smokers used CAM less than nonsmokers.^44^ The data concerning this issue are scarce; however, a systematic review indicated that the effect of various types of CAM on addiction was vague or negative; CAM usage for addiction is unknown, and more studies in this regard are necessary.^45^

### Limitations

Although this study provided vital data concerning the fear of cancer recurrence among cancer survivors, the results of this study should be viewed with limitations. For example, this study used a cross-sectional design that precludes a cause-and-effect relationship, and this made generalization of the results difficult among cancer survivors in a larger population; nonetheless, we believe that our results are robust because the study participants were from different clinical settings such as hospitals, medical centers, and doctor offices. However, a longitudinal prospective study is highly important to infer the definite cause and effect related to the topic at hand. This study also used self-reporting questionnaires that could have increased the recall bias; however, we believe this study has carefully articulated the research questions, and we chose appropriate sample and design. In addition, people who were illiterate received help in filling in the questionnaires, which could have introduced social desirability bias; however, we were aware of this bias; thus, the researcher did not intervene. The researcher only read the questions to the participant and wrote down the answer without any comment.

## Implications on clinical nursing practice

Integrating CAM techniques into patient care at the bedside has the potential to improve the quality of nursing and address the unique needs of patients. The utilization of CAM methods offers several advantages, including their affordability and the minimal requirement for specialized equipment. Moreover, these methods have the potential to create a positive and comforting experience for patients. Nurses can effectively incorporate CAM techniques to provide a diverse range of nursing interventions for the comprehensive care of cancer patients. It is crucial for nurses to be knowledgeable about these methods and to consider their implementation when suitable for the patient's circumstances. By doing so, nurses can contribute to a holistic and patient-centered approach to care.

## Conclusions

The study results indicated that most of the survivors used at least one type of CAM in the previous year, including medicinal herbs, nutritional supplements, meditation, dry cupping, wet cupping, and massage. Patients with cancer must increase their awareness of the effect of CAM. Nurses and other healthcare workers have more contact with patients; thus, their information about the benefits and risks of using CAM can be helpful. We observed that the mean score of FCRI was moderate, and the FCRI and its dimensions did not differ between users and non-users of CAM. We recommend that ongoing counseling services should be available for cancer survivors to help them cope and reduce their fear of recurrence. Hence, policymakers are invited to initiate and establish counseling services that are directed to reduce the fear of cancer recurrence. Health managers and planners should provide psychological interventions and different strategies to reduce the fear of cancer recurrence among cancer survivors. Further studies on other factors that affect the fear of cancer recurrence among cancer survivors are necessary. Cancer survivors require psychosocial support, monitoring of their mental health status, and timely and continuous interventions.

## Data Availability

Data are available by contacting the corresponding author by email.

## CRedit author statement

Mahlagha Dehghan: Conceptualization, Methodology, analysis, writing. Mohammad Ali Zakeri: Data collection, Methodology, Writing - Original draft preparation. Leyla Ahmadi Lari: Data collection, Writing. Heliang Wu & Adnan Rashid Aziz & Rasmieh Al-Amer: Original and revised draft preparation. All authors had full access to all the data in the study, and the corresponding author had final responsibility for the decision to submit for publication. The corresponding author attests that all listed authors meet authorship criteria and that no others meeting the criteria have been omitted.
